# Transduction with BBF2H7/CREB3L2 upregulates SEC23A protein in erythroblasts and partially corrects the hypo‐glycosylation phenotype associated with CDAII

**DOI:** 10.1111/bjh.15189

**Published:** 2018-03-14

**Authors:** Stéphanie Pellegrin, Katy L. Haydn‐Smith, Lea A. Hampton‐O'Neil, Bethan R. Hawley, Kate J. Heesom, Elisa Fermo, Paola Bianchi, Ashley M. Toye

**Affiliations:** ^1^ School of Biochemistry Biomedical Sciences Building University Walk Bristol Bristol UK; ^2^ Bristol Institute of Transfusion Sciences NHSBT Filton Bristol UK; ^3^ National Institute for Health Research (NIHR) Blood and Transplant Unit in Red Blood Cell Products at the University of Bristol Bristol UK; ^4^ Haematology Unit Physiopathology of Anaemia Unit Foundation IRCCS Ca’ Granda Ospedale Maggiore Policlinico Milan Italy

**Keywords:** erythropoiesis, erythroid cell differentiation, hereditary anaemias, congenital dyserythropoietic anaemia II, SEC23

Congenital Dyserythropoietic Anaemia II (CDAII) is an autosomal recessive hereditary anaemia caused by mutations in the *SEC23B* gene (Bianchi *et al*, 2009; Schwarz *et al*, 2009). CDAII erythroid cells are characterized by multi‐nuclearity in 10–50% of mature erythroblasts in the bone marrow; hypo‐glycosylation of membrane proteins and the presence, in a proportion of erythrocytes, of a double cell membrane resulting from retained endoplasmic reticulum (ER) (Heimpel *et al*, 2003; Denecke *et al*, 2008; Satchwell *et al*, 2013). Humans have two SEC23 paralogues, SEC23A and SEC23B, that share 85% amino acid sequence identity. These proteins comprise part of the coat protein complex II (COPII), responsible for anterograde vesicle trafficking from the ER to the cis‐golgi.

Satchwell *et al* (2013) demonstrated that both SEC23 proteins are expressed in human erythroid progenitor cells but SEC23A is rapidly lost during differentiation. This suggests that mutations in the human *SEC23B* gene affect erythroid cells specifically because of a reliance on SEC23B during human erythropoiesis. In mice, the *Sec23b* knockout does not recapitulate the CDAII phenotype (Khoriaty *et al*, 2014), most likely because SEC23A expression is maintained throughout murine erythropoiesis (Satchwell *et al*, 2013). Whether SEC23A can fully compensate for reduced SEC23 function in the presence of mutated SEC23B in human erythroid cells is unknown but Russo *et al* (2013) noted a compensatory increase in *SEC23A* mRNA expression in 2 CDAII patients with a mild phenotype despite low SEC23B expression. We therefore investigated whether up‐regulation of endogenous SEC23A in erythroid cells would compensate for reduced SEC23B function in CDAII erythroid cells.

SEC23A is up‐regulated in mouse chondrocytes in response to the transcriptionally active N‐terminal p60‐fragment of BBF2H7 which binds to the promoter region of *Sec23a* during ER stress (Kondo *et al*, 2007; Saito *et al*, 2009). We first investigated whether introduction of p60‐BBF2H7 would counteract the normal loss of SEC23A protein expression in differentiating primary human erythroid cells of both healthy donors and CDAII patients.

Human p60‐BBF2H7 (residues 1–377) was cloned into the pXLG3‐GFP lentiviral expression vector in frame with the N‐terminal Green Fluorescent protein (GFP) (Figure S1A; Supplemental Methods). The experimental timeline for the culture and transduction of erythroid cells is illustrated in Fig 1B. After 6 days in culture, peripheral blood mononuclear cells (PBMC)‐derived donor erythroblasts were transduced with either pXLG3‐GFP‐p60‐BBF2H7 or pXLG3‐GFP, generating 2 separate cultures for each donor. On day 8, GFP‐positive cells were sorted to obtain pure populations of GFP or GFP‐p60‐BBF2H7 expressing cells (Figure S1B; Supplemental Methods). This protocol was followed for 4 healthy donors and 3 CDAII patients with different *SEC23B* genotypes (E109K/E109K; R190X/S603L or R14W/R554X). Enough erythroblasts were obtained from patient E109K/E109K to repeat this protocol a second time, giving a total of 4 CDAII cultures from 3 patients.

**Figure 1:**
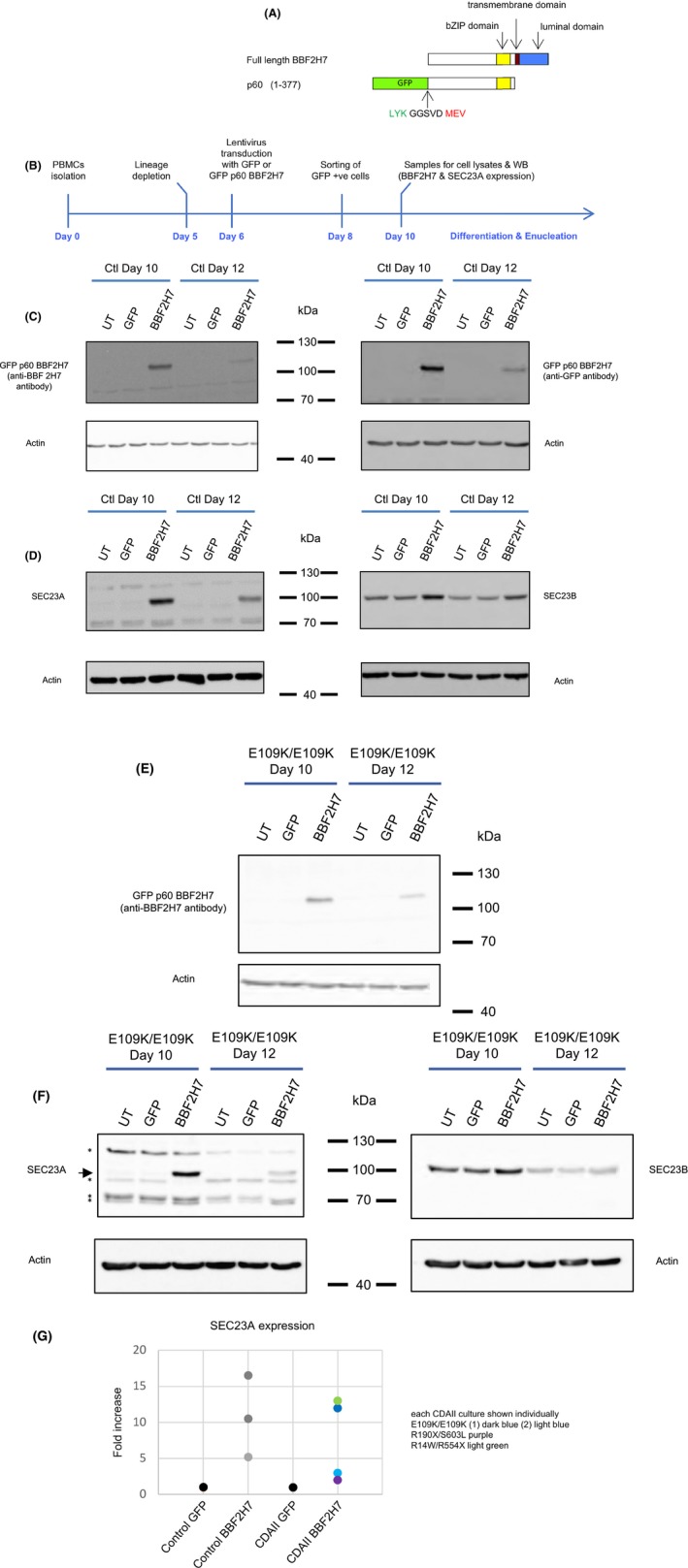
GFP‐p60‐BBF2H7 over‐expression in primary human erythroblasts and up‐regulation of endogenous SEC23A. (A) Cloning of the N‐terminal p60‐fragment of BBF2H7 in a GFP lentiviral vector (GFP‐p60‐BBF2H7). p60 encompasses the first 377 residues of BBF2H7 (human BBF2H7 numbering). The last 3 amino acids of the N‐terminal GFP are shown in green, the vector sequence between the GFP and p60 BBF2H7 is shown in black (5 amino acids: GGSVD) and the first 3 amino acids of p60‐BBF2H7 are shown in red. (B) Protocol and experimental timeline followed for all cultures. (C–D) Western blotting of primary human erythroblast lysates from 3 separate cultures, all derived from the same healthy control donor (un‐transduced, transduced with GFP or GFP‐p60 BBF2H7) using antibodies against BBF2H7 (C, left), GFP (C, right), SEC23A (D, left), SEC23B (D, right) or actin as a loading control (C‐D). The anti‐SEC23A and anti‐SEC23B antibodies have been described previously (Satchwell *et al*, 2013). (E–F) Western blotting of 3 separate cultures (un‐transduced, transduced with GFP or GFP‐p60‐BBF2H7) of primary human erythroblasts lysates all derived from the same CDAII patient [E109K/E109K (culture 1)], using antibodies against BBF2H7 (E), SEC23A (F, left; the arrow points to SEC23A, the asterisks indicate non‐specific bands), SEC23B (F, right) or actin as a loading control. (G) Quantification by densitometry of SEC23A expression on Day 10 from Western blots of healthy control cultures (*n* = 3) and CDAII patients (4 cultures from 3 patients). The y axis values show the ratio of SEC23A over actin, normalised to that of the GFP cultures. For each culture, the value obtained for the GFP cells was set to 1 and the values for the GFP‐p60 BBF2H7 cells show the fold increase when compared to GFP expressing cells from the same donor. The values obtained for the 3 control cultures expressing GFP‐p60 BBF2H7 are shown in different shades of grey. The values obtained for 4 CDAII cultures expressing GFP‐p60 BBF2H7 are for E109K/E109K (culture 1, dark blue); E109K/E109K (culture 2, light blue); R190X/S603L (purple) and R14W/R554X (light green).

Cell samples were taken from all cultures on day 10 to quantify the expression of GFP‐p60‐BBF2H7 and SEC23A using Western blotting. Over‐expression of GFP‐p60‐BBF2H7 was confirmed on day 10 and this decreased by day 12 as the cells differentiated (Fig 1C and E). Concomitant induction of endogenous SEC23A was also observed (Fig 1D and F). Quantification by densitometry showed that SEC23A induction varied between cultures (Fig 1G). On average, SEC23A was increased 10.5‐fold in healthy control cells and 7.5‐fold in CDAII patient cells. SEC23B expression, where measured, was increased by 2‐ and 1.3‐fold in control and CDAII cells respectively (Figure S1). In addition to its up‐regulation in response to GFP‐p60‐BBF2H7, SEC23A expression persisted for longer (Fig 1D and F). These data show for the first time that over‐expression of GFP‐p60‐BBF2H7 can counteract the normal loss of endogenous SEC23A in both normal and CDAII erythroid cells.

We next investigated whether the introduction of GFP‐p60‐BBF2H7 and the persistence of SEC23A had any detectable effect on cultured erythroid cells. The growth curves show that overexpression of GFP‐p60‐BBF2H7 had a partial effect on cell proliferation relative to the GFP‐expressing cells from the same donor (Fig 2A and B). In 1 out of 4 control cultures but 3 out of 4 CDAII cultures, over‐expression of GFP‐p60‐BBF2H7 yielded more cells than expression of GFP alone. Furthermore, over‐expression of GFP‐p60‐BBF2H7 had no obvious effect on erythroblast differentiation (Figures S2 and S3).

**Figure 2:**
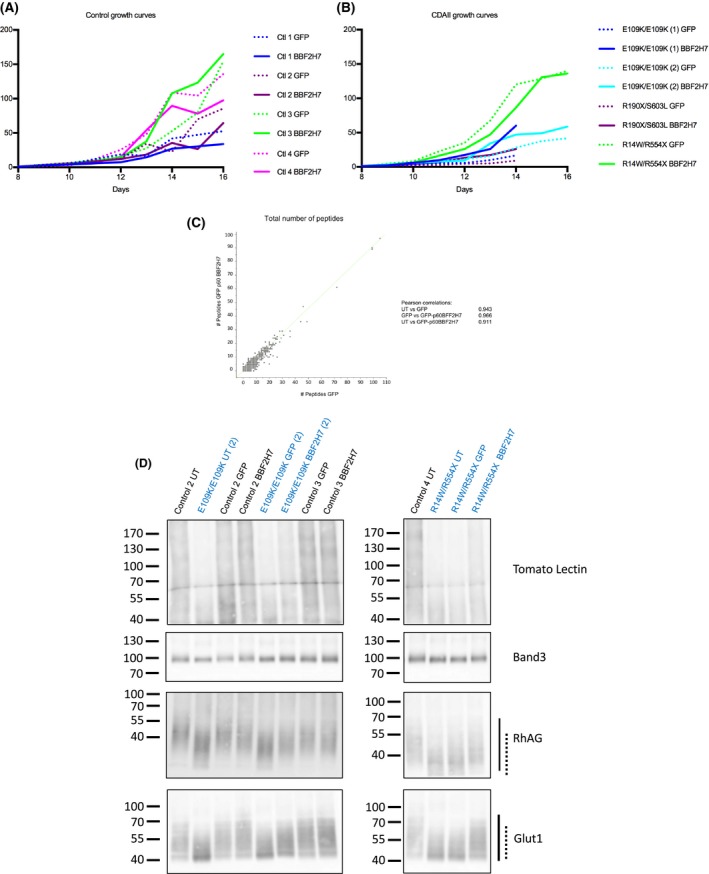
Effects of GFP‐p60‐BBF2H7 expression on *in vitro* erythropoiesis. (A) Growth curves of healthy control cells (*n* = 4). Dotted lines are used for GFP‐expressing cells and same colour solid lines are used for cells from the same donor expressing GFP‐p60 BBF2H7. (B) Growth curves of CDAII patient cells (4 cultures from 3 CDAII patients). Dotted lines are used for GFP expressing cells and same colour solid lines are used for cells from the same donor expressing GFP‐p60 BBF2H7. (C) Scatter plot of label free peptide numbers for each of the 2002 proteins identified by mass spectrometry using 2 × 10^6^ *in vitro* reticulocytes obtained from GFP *versus* GFP‐p60 BBF2H7 expressing erythroblasts, derived from the same healthy control donor. 2 × 10^6^ reticulocytes filtered from each of 3 cultures, derived from the same control healthy donor (un‐transduced, GFP‐expressing or GFP‐p60 BBF2H7‐expressing cells) were used for proteomics analysis of whole cell lysates and the data was analysed using the Perseus software (Max Planck Institute, Planegg, Germany). In total, 2002 proteins were identified, and the Pearson correlation showed that there was no significant difference between any of the 3 cultures (Pearson correlations for UT *versus* GFP: 0.943; GFP *versus* BBF2H7: 0.966; UT *versus* BBF2H7: 0.911). (D) Western blotting of *in vitro* reticulocyte lysates using Tomato Lectin or antibodies against highly glycosylated membrane proteins: Band3, RhAG and Glut1. The overall hypo‐glycosylation of reticulocyte lysates derived from CDAII and control cells (un‐transduced, GFP‐ or GFP‐p60‐BBF2H7 expressing cells) is visualised by using Tomato Lectin (top panel; controls (black writing) and 2 cultures of CDAII patients (blue writing)). Hypo‐glycosylation of specific membrane proteins (i.e. Band3, RhAG or Glut1) is shown below by the protein's mobility shift. Hypo‐glycosylation of RhAG and Glut1 is evident when comparing control to CDAII reticulocyte lysates; RhAG and Glut1 glycosylation is partially restored in CDAII by GFP‐p60 BBF2H7 expression. The solid lines on the side of the blots show the mobility of the glycosylated protein in healthy controls whereas the dotted lines show the mobility of the hypo‐glycosylated form as seen in CDAII cells.

To ascertain whether expression of GFP‐p60‐BBF2H7 in erythroid progenitors altered the reticulocyte proteome, total reticulocyte lysates obtained from un‐transduced, GFP or GFP‐p60‐BBF2H7 transduced erythroblasts were compared using nano LC‐MS/MS mass spectrometry (Supplemental Methods). The total number of peptides for each of the 2002 proteins identified across the 3 reticulocyte samples was compared. The Pearson correlation coefficients (Fig 2C) and the absence of outliers confirmed that the abundance of each protein did not vary between samples.

Finally, we assessed whether overexpression of GFP‐p60‐BBF2H7 could rescue any of the CDAII phenotypes. Cultured reticulocytes, whether grown from healthy or CDAII cells, are immature and retain ER remnants (Satchwell *et al*, 2013), so the double cell membrane observed in CDAII erythrocytes could not be studied. Hypo‐glycosylation of membrane proteins was however analysed in the 2 CDAII cultures that yielded enough reticulocytes. Western blotting with tomato lectin was used to confirm hypo‐glycosylation of CDAII proteins compared to normal controls. We also investigated specific erythroid membrane proteins known to be hypo‐glycosylated in CDAII. It was confirmed that CDAII reticulocyte lysates display hypo‐glycosylated proteins (Fig 2D). The hypo‐glycosylation phenotype was partially rescued by over‐expression of GFP‐p60‐BBF2H7, as judged by the partial change in tomato lectin binding, the subtle mobility shift for Band3 and the more noticeable mobility shifts in the highly glycosylated proteins RhAG and Glut1 (Fig 2D).

In summary, we have demonstrated for the first time that lentiviral transduction of p60‐BBF2H7 into primary human erythroid cells causes SEC23A upregulation and persistence during terminal erythroid differentiation in both normal and CDAII cells. No detrimental effects were observed during the overexpression of p60‐BBF2H7 on erythroblast proliferation, differentiation or on the reticulocyte proteome. In CDAII erythroblasts, the overexpression of p60‐BBF2H7 increased cell numbers in 3 out of 4 cultures and also partially rescued the hypo‐glycosylation defect, most obvious for highly glycosylated erythroid membrane proteins RhAG and Glut1. In accordance with a recent abstract suggesting that SEC23A functionally overlaps with SEC23B (Khoriaty *et al*, 2017), our study on primary human erythroblasts is supportive of therapeutic strategies aimed at up‐regulating SEC23A, such as increasing BBF2H7 expression, to achieve normalisation of SEC23 levels to compensate for the mutated SEC23B in CDAII patients.

## Author contributions

SP and KH‐S were responsible for the majority of the experimental work. LH‐ON and BRH assisted experimental work and LH‐ON prepared samples for proteomics and conducted proteomics analysis. KH ran the proteomics experiment and produced initial analysis. EP and PB performed molecular testing and managed patient samples. AMT was the principal investigator. SP and AMT designed the experiments and wrote the paper. All authors read and edited the final version of the manuscript.

## Supporting information


**Fig S1.** Quantification of SEC23B expression.
**Fig S2.** Differentiation of untransduced, GFP or GFP‐p60‐BBF2H7 expressing healthy control cells.
**Fig S3.** Differentiation of untransduced, GFP or GFP‐p60‐BBF2H7 expressing CDAII cells.
