# Supplementary material for: Transduction with BBF2H7/CREB3L2 upregulates SEC23A protein in erythroblasts and partially corrects the hypo‐glycosylation phenotype associated with CDAII
Source: Br J Haematol. 2018 Mar 14;184(5):876–81. doi: 10.1111/bjh.15189 (PMC6491999; doi:10.1111/bjh.15189)
Supplement: Supplementary file 1 — Fig S1. Quantification of SEC23B expression. Fig S2. Differentiation of untransduced, GFP or GFP‐p60‐BBF2H7 expressing healthy control cells. Fig S3. Differentiation of untransduced, GFP or GFP‐p60‐BBF2H7 expressing CDAII cells. [file BJH-184-876-s001.pdf]

Supplemental Data for:

Transduction with BBF2H7/CREB3L2 upregulates SEC23A protein in erythroblasts and partially corrects the hypo-glycosylation phenotype associated with CDAII

Stéphanie Pellegrin [1,2,3], Katy Haydn-Smith[1,2], Lea Hampton O'Neil[1,3], Bethan R Hawley[1,3], Kate Heesom[1], Elisa Fermo[4], Paola Bianchi[4] and Ashley M. Toye[1,2,3]

1. School of Biochemistry, Biomedical Sciences Building, University Walk Bristol. BS8 1TD
2. Bristol Institute of Transfusion Sciences, NHSBT Filton, Bristol. BS34 7QH
3. National Institute for Health Research (NIHR) Blood and Transplant Unit in Red Blood Cell Products at the University of Bristol. BS8 1TD.
4. Foundation IRCCS Ca' Granda Ospedale Maggiore Policlinico, Hematology Unit, Physiopathology of Anaemia Unit, Via F. Sforza, 35-20122 Milan, Italy

Corresponding author:

Dr Ashley Toye
School of Biochemistry
Biomedical Sciences Building
University Walk
Bristol
BS8 1TD

Tel: 44 (0)117 3312111
Email: ash.m.toye@bristol.ac.uk

Supplemental Materials and Methods

Cloning GFP-p60 BBF2H7

The human p60 BBF2H7 sequence (amino acids 1-377) was ordered from Genscript and cloned into the lentiviral pXLG3-GFP vector using Sal1 and Mlu1 (Figure 1A). p60-BBF2H7 was cloned in frame with the N-terminal GFP tag of the pXLG3-GFP vector to form GFP-p60 BBF2H7. Correct cloning was confirmed by sequencing and the linker sequence between the N-terminal GFP and p60-BBF2H7 is shown in Figure 1A. The pXLG3-GFP vector has been used in previous studies (Satchwell *et al.* 2015). Expression of full-length GFP or GFP-p60-BBF2H7 using these constructs was first confirmed in K562 cells by Western blotting before embarking on this study using primary human erythroid cells.

Donor and patient blood

Waste blood from anonymous platelet apheresis donors (NHSBT, Filton, UK) or patient samples were provided with written informed consent for research use given in accordance with the Declaration of Helsinki. Research was reviewed and approved by Bristol Research Ethics Committee (REC Number 12/SW/0199). Peripheral blood samples (20 mL) were obtained from 3 unrelated CDAII patients with known *SEC23B* gene mutations (E109K/E109K; R190X/S603L; R14W/R554X).

Erythroid cell culture

Erythroblasts were cultured from peripheral blood mononuclear cells (PBMCs) using a three-stage method as described previously (van den Akker et al. 2010; Griffiths et al. 2012; Bell et al. 2013). The experimental timeline for the culture and transduction of erythroid cells is illustrated in Figure 1B.

This protocol was followed for 4 different healthy donors and 3 CDAII patients with different *SEC23B* genotypes (E109K/E109K; R190X/S603L or R14W/R554X). Enough erythroblasts were obtained from patient E109K/E109K to repeat this protocol a second time, giving a total of 4 CDAII cultures from 3 individual patients (called R190X/S603L, R14W/R554X, E109K/E109K (1), E109K/E109K (2)).

PBMCs were seeded in first stage medium, consisting of the base medium [IMDM (Source BioScience) containing 3% (v/v) AB Serum (Sigma), 2% (v/v) foetal bovine serum (Hyclone, Fisher Scientific UK Ltd), 10µg/ml Insulin (Sigma), 3U/ml heparin (Sigma) and 200µg/ml Transferrin (R&D Systems)], supplemented with 10ng/ml SCF, 1ng/ml IL-3 and 3U/ml EPO. On Day 5, a lineage depletion step (Lineage Cell Depletion Kit, Miltenyi Biotec, UK) was performed to ensure complete removal of lineage positive cells. On Day 6, erythroid progenitors were lentivirally transduced with GFP-p60-BBF2H7 or GFP alone as control (empty pXLG3-GFP vector). On Day 8, GFP positive cells were isolated by Fluorescence Activated Cell Sorting (FACS) for both GFP and GFP-p60 BBF2H7 cultures using a BD Influx Cell Sorter (Figure 1B). After sorting, the GFP positive cells were seeded in second phase medium [base medium supplemented with 10ng/mL SCF, 3U/mL EPO and additional 300µg/ml iron saturated transferrin]. From Day 9 onwards, cells were counted daily and fed accordingly. Growth curves (cumulative cell numbers) were obtained from the daily counts. On day 10, cells were harvested for Western blotting analysis of GFP-p60-BBF2H7, SEC23A and when possible (depending on cell numbers) SEC23B expression. On Day 11, cells were fed using the third stage medium [base medium supplemented with 3U/mL EPO and additional 300µg/ml iron saturated transferrin]. Reticulocytes were filtered on Day 16 using 3µm pore sized Corning Transwells for 6-well plates.

Due to the small volume of starting material and to the fact that erythroid cells from each CDAII donor were divided into 3 parallel cultures (untransduced, GFP and GFP-p60-BBF2H7 expressing cells), the total cell number for each culture was relatively low. For 2 out of the 4 CDAII cultures, a large proportion of early cells (basophilic normoblasts) was used to carry out an extensive study of GFP-p60 BBF2H7, SEC23A and SEC23B expression and therefore very few reticulocytes were obtained. The hypo-glycosylation of reticulocyte membrane proteins was only thoroughly analysed in the other 2 CDAII cultures where fewer early cells (basophilic normoblasts) were removed from the culture for Western blotting analysis and therefore more reticulocytes were obtained.

Cytospins

Cells were cytospun onto glass slides, fixed in methanol for 15 min and stained with May-Grünwald-Giemsa stains according to the manufacturer's protocol. Images were taken with a Leica DM750 microscope coupled to a Pixera Penguin 600CL camera using a 40x lens. For each cytospin, at least 200 cells were scored.

Western Blotting

Cells were lysed for 10 min on ice in lysis buffer (20 mM Tris-HCl, pH 8.0, 137 mM NaCl, 10 mM EDTA, 100 mM NaF, 1% (v/v) Nonidet P-40, 10% (v/v) glycerol, 10 mM Na_3VO_4, 2 mM PMSF and protease inhibitors, Calbiochem), followed by centrifugation at 4^oC at 16,000g for 10 min.

For Western blotting of Day 10 or Day 12 erythroblast lysates, the equivalent of 0.5x10e6 cells were loaded into each well, separated by SDS- polyacrylamide gel electrophoresis and transferred to PVDF membrane. The primary antibodies used were against GFP (Roche), BBF2H7/CREB3L2 (ProteinTech), SEC23A or SEC23B (in house antibodies, described previously in Satchwell et al., 2013) and Actin (Sigma) as a loading control. For glycosylation studies of filtered reticulocyte lysates, 10µg of lysate were loaded into each well. For tomato lectin blots, the PVDF membranes were blocked for 1 hour in 1x Carbo-free Blocking solution (Vector Laboratories). Blots were incubated for 45 min with biotinylated tomato lectin (Vector Laboratories, used at 1µg/ml), washed three times in TBST and incubated 45 min with ExtrAvidin HRP (Sigma).

For mobility shift studies (analysis of membrane protein hypo-glycosylation), antibodies against Band3 (monoclonal BRic170), RhAG (rabbit polyclonal YUDY) and Glut1 (rabbit polyclonal antibody; gift from Dr Lesley Bruce, NHSBT Filton, Bristol, UK) were used. Secondary antibodies were HRP-conjugated rabbit-anti–mouse and swine anti–rabbit (Dako). The signals were detected using ECL detection reagent (GE Healthcare) and the Amersham Imager 600 (GE Healthcare).

Quantification by densitometry of SEC23A or SEC23B expression was carried out using the Image Quant TL software (GE Healthcare). For each lane, the ratio of the intensity of the SEC23A or SEC23B signal over that of the loading control actin was calculated. The values obtained for each culture of GFP-expressing cells was set to 1 and the fold increase for the GFP-p60-BBF2H7 expressing cells from the same donor was calculated. Values obtained for SEC23A-fold increase in individual cultures are shown in Figure 1G. The mean of these values was calculated as 10.5-fold increase in healthy control cells (n=3) and 7.5-fold increase in CDAII patient cells (4 cultures from 3 different CDAII patients) for SEC23A protein expression. SEC23B expression, where measured, was increased by 2 and 1.3-fold in control and CDAII cells respectively (Figure S1).

Flow Cytometry

To monitor erythroid differentiation, $1x10^5$ cells were labelled with directly-conjugated antibodies against cell surface markers (anti-CD36 PE and anti-GPA APC, Miltenyi Biotech) or isotype-matched IgG control antibodies (Miltenyi Biotech) as described previously (Severn et al., 2016). Enucleation was quantified as described previously (Satchwell et al, 2015). Briefly, $1x10^5$ cells were stained for 30 min at 37°C with 5 μg/mL Hoechst 33342 (Life Technologies). Data were collected using a MacsQuant flow cytometer (Miltenyi Biotec) and processed using FlowJo Version 10.

Proteomics

In order to determine whether expression of GFP-p60-BBF2H7 in erythroid progenitors resulted in changes in the reticulocyte proteome, total reticulocyte lysates were compared using nano LC-MS/MS mass spectrometry. *In vitro* reticulocytes grown from untransduced (UT), GFP or GFP-p60 BBF2H7 transduced erythroblasts from the same healthy donor were filtered using 3μm pore sized Corning transwells for 6-well plates and $2x10^6$ filtered reticulocytes from each of the 3 cultures (UT, GFP or GFP-p60 BBF2H7) were lysed and used for proteomics analysis.

The samples were run approximately 2cm into a 10% separating gel and the gel lane then cut into 2 equal slices and each slice subjected to in-gel tryptic digestion using a DigestPro automated digestion unit (Intavis Ltd.). The resulting peptides were fractionated using an Ultimate 3000 nanoHPLC system in line with an LTQ-Orbitrap Velos mass spectrometer (Thermo Scientific). In brief, peptides in 1% (vol/vol) formic acid were injected onto an Acclaim PepMap C18 nano-trap column (Thermo Scientific). After washing with 0.5% (vol/vol) acetonitrile 0.1% (vol/vol) formic acid peptides were resolved on a 250 mm × 75 μm Acclaim PepMap C18 reverse phase analytical column (Thermo Scientific) over a 150 min organic gradient, using 7 gradient segments (1-6% solvent B over 1min., 6-15% B over 58min., 15-32%B over 58min., 32-40%B over 5min., 40-90%B over 1min., held at 90%B for 6min and then reduced to 1%B over 1min.) with a flow rate of 300 nl min^{-1}. Solvent A was 0.1% formic acid and Solvent B was aqueous 80% acetonitrile in 0.1% formic acid. Peptides were ionized by nano-electrospray ionization at 2.1 kV using a stainless-steel emitter with an internal diameter of 30 μm (Thermo Scientific) and a capillary temperature of 250°C. Tandem mass spectra were acquired using an LTQ- Orbitrap Velos mass spectrometer controlled by Xcalibur 2.1 software (Thermo Scientific) and operated in data-dependent acquisition mode. The Orbitrap was set to analyze the survey scans at 60,000 resolution (at m/z 400) in the mass range m/z 300 to 2000 and the top twenty multiply charged ions in each duty cycle selected for MS/MS in the LTQ linear ion trap. Charge state filtering, where unassigned precursor ions were not selected for fragmentation, and dynamic exclusion (repeat count, 1; repeat duration, 30s; exclusion list size, 500) were used.

Fragmentation conditions in the LTQ were as follows: normalized collision energy, 40%; activation q, 0.25; activation time 10ms; and minimum ion selection intensity, 500 counts.

The raw data files were processed and quantified using Proteome Discoverer software v1.4 (Thermo Scientific) and searched against the UniProt Human database (126385 sequences) using the SEQUEST algorithm. Peptide precursor mass tolerance was set at 10ppm, and MS/MS tolerance was set at 0.8Da. Search criteria included carbamidomethylation of cysteine (+57.0214) as a fixed modification and oxidation of methionine (+15.9949) as a variable modification. Searches were performed with full tryptic digestion and a maximum of 1 missed cleavage was allowed. The reverse database search option was enabled and all peptide data was filtered to satisfy false discovery rate (FDR) of 5%.

The data was analysed using the Perseus software (Max Planck Institute, Germany). A total of 2002 proteins were identified across the 3 samples and the total number of peptides for each protein, used as an indicator of protein abundance, was compared. The Pearson correlation coefficient, which is a measure of the linear correlation between two variables X and Y, is very close to 1 for each of the 3 comparisons (UT vs GFP: 0.943; GFP vs BBF2H7: 0.966; UT vs BBF2H7: 0.911), indicating that expression of GFP-p60-BBF2H7 did not affect the reticulocyte proteome (Figure 2C). These data were also examined for outliers and none were identified, confirming that between samples, the abundance of each protein did not vary noticeably.

Supplemental References

Bell AJ, Satchwell TJ, Heesom KJ, Hawley BR, Kupzig S, Hazell M, Mushens R, Herman A, Toye AM. Protein distribution during human erythroblast enucleation in vitro. PLoS One. 2013;8(4):e60300.

Griffiths RE, Kupzig S, Cogan N, Mankelow TJ, Betin VM, Trakarnsanga K, Massey EJ, Lane JD, Parsons SF, Anstee DJ. Maturing reticulocytes internalize plasma membrane in glycophorin A-containing vesicles that fuse with autophagosomes before exocytosis. Blood. 2012 Jun 28;119(26):6296-306.

Satchwell TJ, Hawley BR, Bell AJ, Ribeiro ML, Toye AM.
The cytoskeletal binding domain of band 3 is required for multiprotein complex formation and retention during erythropoiesis. Haematologica. 2015 Jan;100(1):133-42.

Severn CE, Macedo H, Eagle MJ, Rooney P, Mantalaris A, Toye AM. Polyurethane scaffolds seeded with CD34(+) cells maintain early stem cells whilst also facilitating prolonged egress of haematopoietic progenitors. Sci Rep. 2016 Aug 30;6:32149.

van den Akker E, Satchwell TJ, Pellegrin S, Daniels G, Toye AM. The majority of the in vitro erythroid expansion potential resides in CD34(-) cells, outweighing the contribution of CD34(+) cells and significantly increasing the erythroblast yield from peripheral blood samples. Haematologica. 2010 Sep;95(9):1594-8.

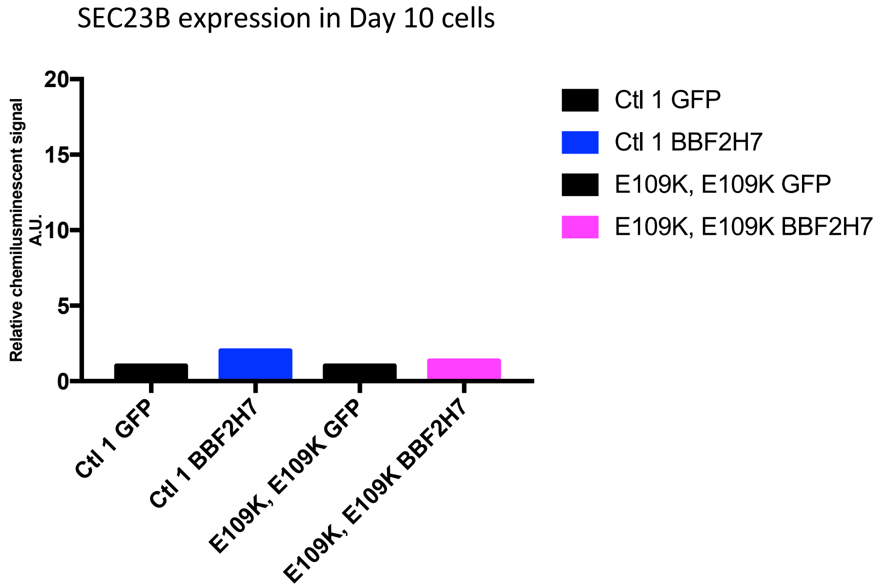

Figure S1:
Quantification of SEC23B expression

Quantification by densitometry of SEC23B expression on Day 10 from Western blots shown in Figure 1 of healthy control and CDAII patient (E109K/E109K (1)) cultures, carried out using the Amersham Imager 600 (GE Healthcare) and the Image Quant TL software (GE Healthcare).

The y axis values show the ratio of SEC23B over actin, normalised to that of the GFP cultures. For each culture, the value obtained for the GFP cells was set to 1 and the values for the GFP-p60 BBF2H7 expressing cells show the fold increase when compared to GFP cells from the same donor.

When comparing cells from the same donor transduced with either GFP-p60-BBF2H7 or GFP alone, SEC23B showed a 2-fold and 1.3-fold increase in control and CDAII cells respectively, whereas SEC23A showed a 16.5-fold and 12-fold increase in the same control and CDAII cells respectively (Figure 1).

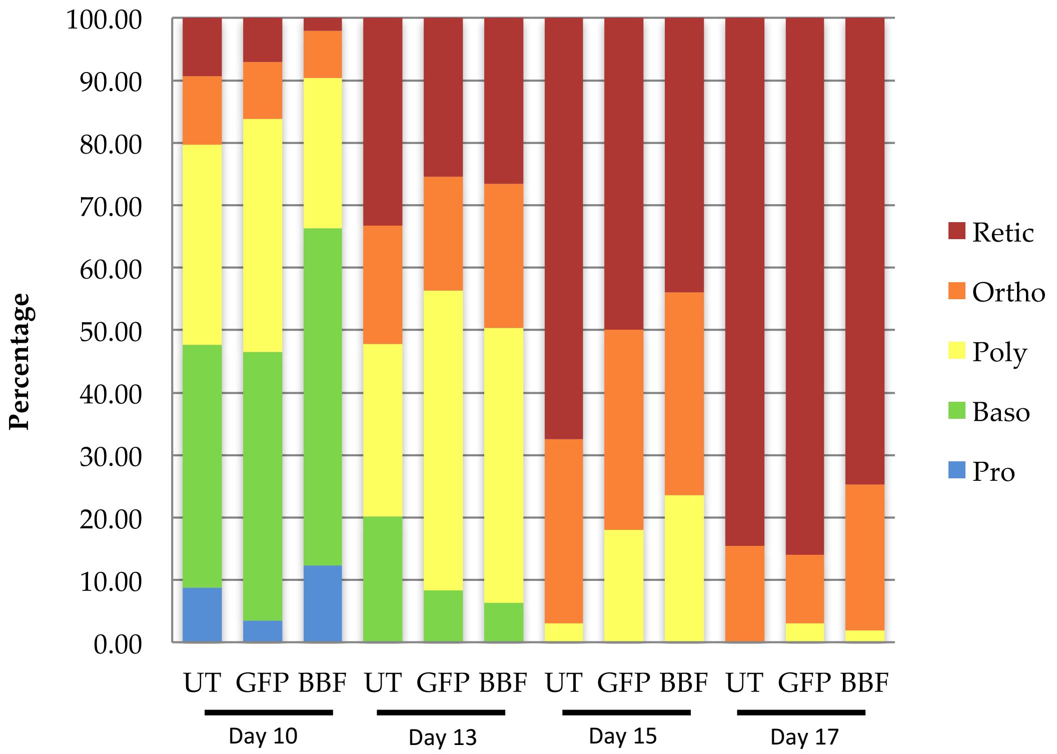

Figure S2:
Differentiation of untransduced, GFP or GFP-p60-BBF2H7 expressing healthy control cells.

Scoring of cytospins from the 3 different cultures derived from the same donor (untransduced (UT), GFP or GFP-p60-BBF2H7 expressing cells) shows that expression of GFP-p60-BBF2H7 does not affect erythroid cells differentiation and does not delay differentiation.

The cells scored here by cytospins are the healthy control cells used for Figure 1 and Figure S1 for which quantification of SEC23A and SEC23B expression by densitometry of Western blots showed that on Day 10, SEC23A was increased 16.5-fold and Sec23B 2-fold when comparing GFP to GFP-p60-BBF2H7 cells.

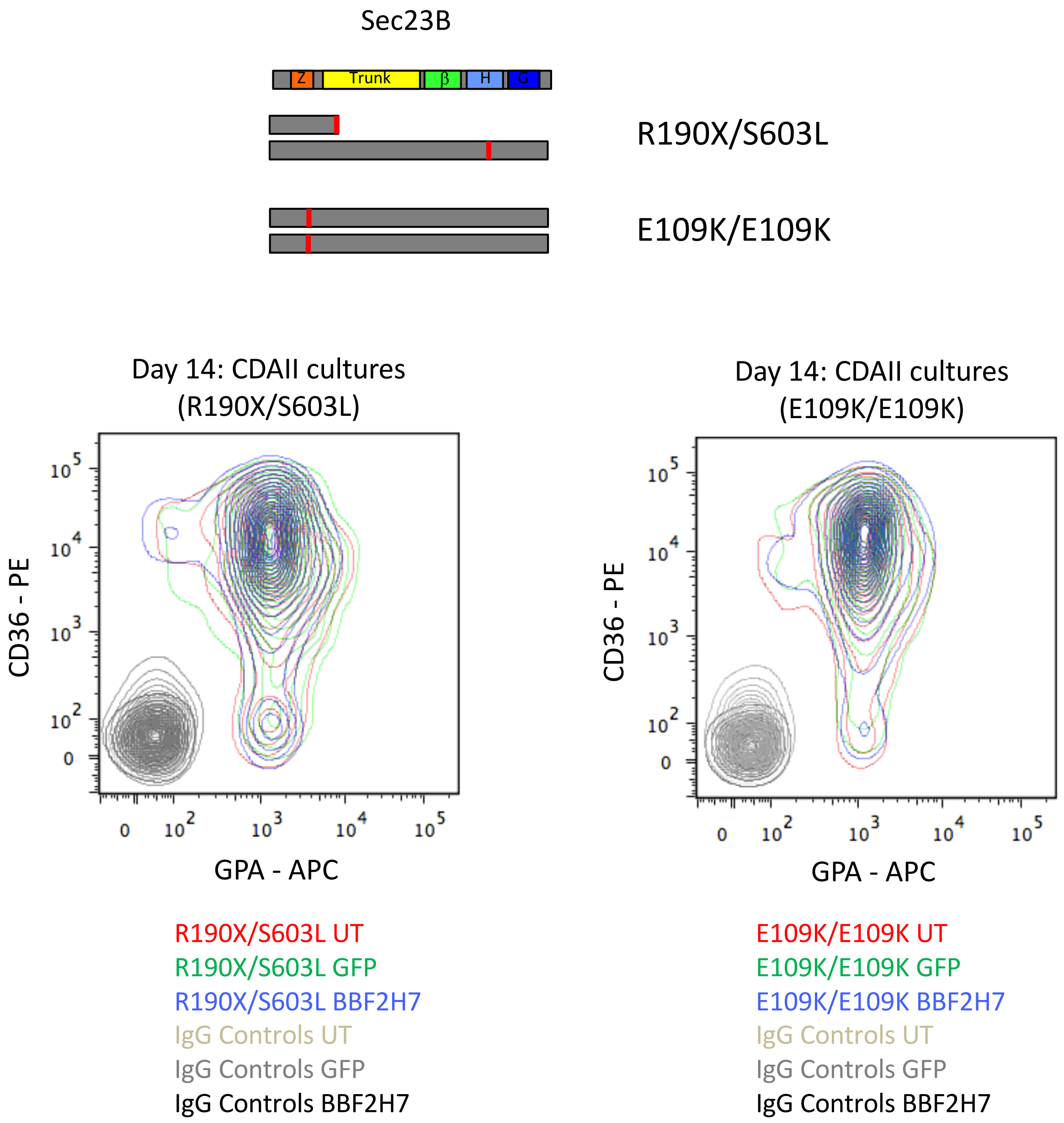

Figure S3:
Differentiation of untransduced, GFP or GFP-p60-BBF2H7 expressing CDAII cells.

Differentiation of the 3 different cultures derived from the same donor (untransduced (UT), GFP or GFP-p60-BBF2H7 expressing cells), grown from 2 different CDAII patients (R190X/S603L and E109K/E109K; the SEC23B mutations are shown schematically in the top panel), was assessed by flow cytometry on Day 14 using antibodies against CD36 and GPA. All 3 cultures exhibit the same differentiated phenotype, indicating that expression of GFP-p60-BBF2H7 does not delay erythroid differentiation, when compared to GFP-expressing or untransduced cells derived from the same donor.
